# Supplementary material for: Neural, physiological and behavioural correlates of empathy for pain in Tourette syndrome
Source: Brain Commun. 2023 Aug 11;5(4):fcad212. doi: 10.1093/braincomms/fcad212 (PMC10438210; doi:10.1093/braincomms/fcad212)
Supplement: fcad212_Supplementary_Data [file fcad212_supplementary_data.pdf]

Neural, physiological and behavioral correlates of empathy for pain in Tourette syndrome

Ronja Weiblen, Carina Robert, Pauline Petereit, Marcus Heldmann, Thomas F. Münte, Alexander Münchau, Kirsten Müller-Vahl, Ulrike M. Krämer

Supplementary Materials

Abbreviations & Factors Overview

ges = generalized eta squared

TS = Tourette Syndrome

HC = Healthy Controls

Explanation of factors:

- Group: TS & HC
- Action: painful & neutral (pictures shown during paradigm)
- Sensitivity: normal & enhanced (pain sensitivity of actor in pictures)
- Perspective: own pain & other pain (who´s pain is rated after the picture?)
- Region: central & occipital (electrode location for EEG)
- Laterality: middle & left & right (electrode location for EEG)

Behavioral Results Empathy for Pain Task

Supplementary table 1: Summary statistics of pain ratings per combination of conditions:

Group	Action	Sensitivity	Perspective	n	mean	sd
HC	neutral	normal	Own pain	25	22.51	21.09
HC	neutral	enhanced	Own pain	25	23.88	20.18
HC	painful	normal	Own pain	25	66.52	15.14
HC	painful	enhanced	Own pain	25	65.50	14.69
TS	neutral	normal	Own pain	21	16.39	10.87
TS	neutral	enhanced	Own pain	21	19.09	14.07
TS	painful	normal	Own pain	21	64.43	18.92
TS	painful	enhanced	Own pain	21	61.57	20.88
HC	neutral	normal	Other pain	25	22.74	21.14
HC	neutral	enhanced	Other pain	25	41.66	23.95
HC	painful	normal	Other pain	25	66.39	14.92
HC	painful	enhanced	Other pain	25	74.63	13.92
TS	neutral	normal	Other pain	21	18.30	13.60
TS	neutral	enhanced	Other pain	21	31.35	16.03
TS	painful	normal	Other pain	21	65.71	15.66
TS	painful	enhanced	Other pain	21	69.62	16.17

Supplementary table 2: Anova with factors Group, Action, Sensitivity and Perspective:

Effect	DFn	DFd	F	p	p<.05	ges
Group	1	44	1.696	0.200		0.02
Action	1	44	203.106	0.000	*	0.61
Sensitivity	1	44	18.799	0.000	*	0.03
Perspective	1	44	29.732	0.000	*	0.03
Group:Action	1	44	0.345	0.560		0.00
Group:Sensitivity	1	44	1.098	0.300		0.00
Group:Perspective	1	44	0.143	0.707		0.00
Action:Sensitivity	1	44	14.255	0.000	*	0.01
Action:Perspective	1	44	11.69	0.001	*	0.00
Sensitivity:Perspective	1	44	47.013	0.000	*	0.03
Group:Action:Sensitivity	1	44	0.049	0.826		0.00
Group:Action:Perspective	1	44	1.057	0.310		0.00
Group:Sensitivity:Perspective	1	44	2.292	0.137		0.00
Action:Sensitivity:Perspective	1	44	11.25	0.002	*	0.00
Group:Action:Sensitivity:Perspective	1	44	1.764	0.191		0.00

Supplementary table 3: Anova for ratings of other's pain:

Effect	DFn	DFd	F	p	p<.05	ges
Group	1	44	1.95	0.170		0.02
Action	1	44	187.46	0.000	*	0.58
Sensitivity	1	44	36.83	0.000	*	0.09
Group:Action	1	44	0.58	0.449		0.00
Group:Sensitivity	1	44	1.97	0.167		0.01
Action:Sensitivity	1	44	21.69	0.000	*	0.02
Group:Action:Sensitivity	1	44	0.13	0.720		0.00

Supplementary table 4: Pairwise Comparisons *Group* per Action and Sensitivity:

Action	Sensitivity	variable	group1	group2	n1	n2	p
neutral	normal	Other pain	HC	TS	25	21	0.412
neutral	enhanced	Other pain	HC	TS	25	21	0.100
painful	normal	Other pain	HC	TS	25	21	0.882
painful	enhanced	Other pain	HC	TS	25	21	0.265

Supplementary table 5: Pairwise Comparisons *Action* per Sensitivity:

Sensitivity	variable	group1	group2	n1	n2	p
normal	Other pain	neutral	painful	46	46	0.000
enhanced	Other pain	neutral	painful	46	46	0.000

Supplementary table 6: Pairwise Comparisons *Sensitivity* per Action:

Action	variable	group1	group2	n1	n2	p
neutral	Other pain	normal	enhanced	46	46	0.000
painful	Other pain	normal	enhanced	46	46	0.049

Supplementary table 7: Anova for ratings of own supposed pain:

Effect	DFn	DFd	F	p	p<.05	ges
Group	1	44	1.19	0.281		0.02
Action	1	44	207.30	0.000	*	0.62
Sensitivity	1	44	0.00	0.965		0.00
Group:Action	1	44	0.16	0.691		0.00
Group:Sensitivity	1	44	0.01	0.910		0.00
Action:Sensitivity	1	44	4.16	0.047	*	0.00
Group:Action:Sensitivity	1	44	0.66	0.422		0.00

Supplementary table 8: Pairwise Comparisons *Sensitivity* per Group and Action:

Group	Action	variable	group1	group2	n1	n2	p
HC	neutral	Own pain	normal	enhanced	25	25	0.815
HC	painful	Own pain	normal	enhanced	25	25	0.810
TS	neutral	Own pain	normal	enhanced	21	21	0.490
TS	painful	Own pain	normal	enhanced	21	21	0.645

EEG Results in Empathy for Pain Task

Supplementary table 9: Summary statistics for mu/alpha power:

Group	Sensitivity	Action	Region	Laterality	n	mean	sd
HC	normal	neutral	central	left	25	-2.22	1.74
HC	normal	neutral	central	middle	25	-1.99	1.74
HC	normal	neutral	central	right	25	-2.03	2.17
HC	enhanced	neutral	central	left	25	-2.44	2.22
HC	enhanced	neutral	central	middle	25	-2.43	2.01
HC	enhanced	neutral	central	right	25	-2.43	2.21
HC	normal	painful	central	left	25	-2.40	2.14
HC	normal	painful	central	middle	25	-2.50	2.13
HC	normal	painful	central	right	25	-2.71	2.03
HC	enhanced	painful	central	left	25	-2.52	1.96
HC	enhanced	painful	central	middle	25	-2.26	1.85
HC	enhanced	painful	central	right	25	-2.42	2.12
TS	normal	neutral	central	left	19	-1.64	1.66
TS	normal	neutral	central	middle	19	-1.57	1.50
TS	normal	neutral	central	right	20	-1.74	2.09
TS	enhanced	neutral	central	left	20	-1.63	1.90
TS	enhanced	neutral	central	middle	20	-1.49	1.95
TS	enhanced	neutral	central	right	20	-1.49	1.59
TS	normal	painful	central	left	20	-2.08	2.00
TS	normal	painful	central	middle	19	-1.55	1.62
TS	normal	painful	central	right	20	-1.69	1.79
TS	enhanced	painful	central	left	20	-1.99	1.83
TS	enhanced	painful	central	middle	20	-1.96	2.05
TS	enhanced	painful	central	right	20	-1.49	1.41
HC	normal	neutral	occipital	left	25	-2.85	2.07
HC	normal	neutral	occipital	middle	25	-2.02	2.39
HC	normal	neutral	occipital	right	25	-2.76	2.58
HC	enhanced	neutral	occipital	left	25	-2.85	2.36
HC	enhanced	neutral	occipital	middle	25	-2.19	2.82
HC	enhanced	neutral	occipital	right	25	-3.07	3.16
HC	normal	painful	occipital	left	25	-3.03	2.38
HC	normal	painful	occipital	middle	25	-2.64	2.61
HC	normal	painful	occipital	right	25	-3.51	2.67
HC	enhanced	painful	occipital	left	25	-3.26	2.48
HC	enhanced	painful	occipital	middle	25	-2.87	2.46
HC	enhanced	painful	occipital	right	25	-3.44	2.88
TS	normal	neutral	occipital	left	20	-3.10	2.66
TS	normal	neutral	occipital	middle	20	-2.30	2.47
TS	normal	neutral	occipital	right	20	-3.73	2.84
TS	enhanced	neutral	occipital	left	20	-2.86	2.25
TS	enhanced	neutral	occipital	middle	20	-1.79	2.21
TS	enhanced	neutral	occipital	right	20	-2.76	2.59
TS	normal	painful	occipital	left	20	-3.27	2.60

TS	normal	painful	occipital	middle	20	-2.49	2.04
TS	normal	painful	occipital	right	20	-3.35	2.53
TS	enhanced	painful	occipital	left	20	-3.52	2.58
TS	enhanced	painful	occipital	middle	20	-2.48	2.45
TS	enhanced	painful	occipital	right	20	-3.40	2.65

Supplementary table 10: Anova with factors Group, Sensitivity, Action, Region and Laterality:

Effect	DFn	DFd	F	p	p<.05	ges
Group	1	41	1.06	0.310		0.02
Sensitivity	1	41	0.04	0.848		0.00
Action	1	41	9.47	0.004	*	0.01
Region	1	41	13.84	0.001	*	0.04
Laterality	2	82	8.51	0.000	*	0.01
Group:Sensitivity	1	41	0.84	0.364		0.00
Group:Action	1	41	0.09	0.764		0.00
Group:Region	1	41	2.31	0.136		0.01
Group:Laterality	2	82	0.15	0.865		0.00
Sensitivity:Action	1	41	0.27	0.608		0.00
Sensitivity:Region	1	41	0.36	0.552		0.00
Action:Region	1	41	4.56	0.039	*	0.00
Sensitivity:Laterality	2	82	1.59	0.211		0.00
Action:Laterality	2	82	0.75	0.476		0.00
Region:Laterality	2	82	8.68	0.000	*	0.01
Group:Sensitivity:Action	1	41	5.31	0.026	*	0.00
Group:Sensitivity:Region	1	41	0.95	0.336		0.00
Group:Action:Region	1	41	0.27	0.605		0.00
Group:Sensitivity:Laterality	2	82	0.81	0.446		0.00
Group:Action:Laterality	2	82	2.18	0.120		0.00
Group:Region:Laterality	2	82	0.68	0.507		0.00
Sensitivity:Action:Region	1	41	3.84	0.057		0.00
Sensitivity:Action:Laterality	2	82	1.00	0.372		0.00
Sensitivity:Region:Laterality	2	82	0.60	0.549		0.00
Action:Region:Laterality	2	82	0.92	0.403		0.00
Group:Sensitivity:Action:Region	1	41	0.30	0.587		0.00
Group:Sensitivity:Action:Laterality	2	82	2.49	0.089		0.00
Group:Sensitivity:Region:Laterality	2	82	1.58	0.213		0.00
Group:Action:Region:Laterality	2	82	0.28	0.756		0.00
Sensitivity:Action:Region:Laterality	2	82	1.14	0.325		0.00
Group:Sensitivity:Action:Region:Laterality	2	82	3.37	0.039	*	0.00

Supplementary table 11: Anova results grouped by Region:

Region	Effect	DFn	DFd	F	p	p<.05	ges
central	Group	1	41	3.36	0.074		0.06
central	Action	1	41	2.87	0.098		0.00
central	Sensitivity	1	41	0.22	0.641		0.00

central	Laterality	2	82	0.95	0.392		0.00
central	Group:Action	1	41	0.00	0.981		0.00
central	Group:Sensitivity	1	41	0.26	0.616		0.00
central	Group:Laterality	2	82	1.03	0.361		0.00
central	Action:Sensitivity	1	41	0.52	0.475		0.00
central	Action:Laterality	2	82	0.00	0.997		0.00
central	Sensitivity:Laterality	2	82	0.09	0.913		0.00
central	Group:Action:Sensitivity	1	41	6.52	0.014	*	0.00
central	Group:Action:Laterality	2	82	2.10	0.129		0.00
central	Group:Sensitivity:Laterality	2	82	0.13	0.876		0.00
central	Action:Sensitivity:Laterality	2	82	2.02	0.140		0.00
central	Group:Action:Sensitivity:Laterality	2	82	1.82	0.169		0.00
occipital	Group	1	43	0.00	0.948		0.00
occipital	Action	1	43	13.80	0.001	*	0.01
occipital	Sensitivity	1	43	0.12	0.735		0.00
occipital	Laterality	2	86	13.73	0.000	*	0.02
occipital	Group:Action	1	43	0.61	0.440		0.00
occipital	Group:Sensitivity	1	43	2.09	0.156		0.00
occipital	Group:Laterality	2	86	0.52	0.598		0.00
occipital	Action:Sensitivity	1	43	2.73	0.106		0.00
occipital	Action:Laterality	2	86	1.23	0.299		0.00
occipital	Sensitivity:Laterality	2	86	1.36	0.262		0.00
occipital	Group:Action:Sensitivity	1	43	3.14	0.083		0.00
occipital	Group:Action:Laterality	2	86	1.88	0.160		0.00
occipital	Group:Sensitivity:Laterality	2	86	1.57	0.215		0.00
occipital	Action:Sensitivity:Laterality	2	86	0.05	0.947		0.00
occipital	Group:Action:Sensitivity:Laterality	2	86	2.81	0.066		0.00

Supplementary table 12: Anova results for central regions, grouped by Group:

Group	Effect	DFn	DFd	F	p	p<.05	ges
HC	Action	1	24	1.56	0.224		0.00
HC	Sensitivity	1	24	0.67	0.421		0.00
HC	Laterality	2	48	0.23	0.798		0.00
HC	Action:Sensitivity	1	24	5.11	0.033	*	0.00
HC	Action:Laterality	2	48	1.19	0.314		0.00
HC	Sensitivity:Laterality	2	48	0.24	0.785		0.00
HC	Action:Sensitivity:Laterality	2	48	2.59	0.085		0.00
TS	Action	1	17	1.48	0.241		0.00
TS	Sensitivity	1	17	0.00	0.983		0.00
TS	Laterality	2	34	2.33	0.113		0.01
TS	Action:Sensitivity	1	17	2.24	0.153		0.00
TS	Action:Laterality	1.5	25.53	0.95	0.377		0.00
TS	Sensitivity:Laterality	2	34	0.00	0.998		0.00
TS	Action:Sensitivity:Laterality	2	34	1.44	0.252		0.00

Supplementary table 13: Pairwise comparisons of action per sensitivity and group:

Group	Sensitivity	variable	group1	group2	n1	n2	p
HC	normal	value	neutral	painful	75	75	0.165
HC	enhanced	value	neutral	painful	75	75	0.917
TS	normal	value	neutral	painful	58	59	0.695
TS	enhanced	value	neutral	painful	60	60	0.399

Skin Conductance Analyses

SCR recording

Skin conductance response (SCR) was recorded using two electrodes attached to the medial phalanges of the index and middle fingers of the left hand. The online reference was placed on the left forearm and recorded with the same amplifier (BrainAmp ExG. BrainProdcts GmbH). As SCR data were measured simultaneously with EEG data, the sampling rate was 500 Hz, and an online high-pass filter of 0.016 Hz as well as a notch filter of 50 Hz were applied.

SCR data analysis

Analysis of skin conductance was performed using the MATLAB® toolbox *Ledalab*[1]. The signal was downsampled to 25 Hz and then smoothed with a Gaussian low-pass filter. Obvious artifacts were removed manually. Then, the signal was deconvolved using a continuous decomposition analysis (CDA) with four optimizations. This separates the signal into continuous traces of tonic and phasic activity. We proceeded to analyze the phasic driver component, by extracting averaged event-related responses via area under the curve, between 1 to 4 seconds after stimulus onset. Event-related phasic SCR were range normalized and log transformed across all trials within each subject. This ensured that individual variability in the signal magnitude would not bias further analyses. SCR per trial were then averaged within conditions. One participant from each group was excluded, as the data quality did not allow measurements of any SCR. Thus, analyses of SCR data were conducted on n = 20 TS patients and n = 24 healthy controls. SCR were analyzed similarly to the EEG data with a mixed ANOVA with the factors group, action and sensitivity. For correlation analyses, difference scores were calculated analogous to the behavioral and EEG data.

SCR results

Analysis of the skin conductance response revealed a significant interaction of group, action and sensitivity ($F(1.42) = 5.40$. $p = .025$. $\eta^2 = .01$; Supplementary figure 1). ANOVAs per group revealed a main effect of action in the TS group ($F(1.19) = 4.39$. $p = .050$. $\eta^2 = .01$), with participants showing an enhanced skin conductance response after watching painful actions. For the healthy controls we found an interaction of action and sensitivity ($F(1.23) = 6.24$. $p = .020$. $\eta^2 = .01$), with nominally stronger responses after painful pictures in the enhanced sensitivity condition. Pairwise comparisons were not significant.

Discussion of SCR results

Physiologically, increased distress due to increased sharing should have been visible in a stronger skin conductance response in the TS group. However, we observed no such group differences. In fact, the results suggested a slightly reduced modulation of skin conductance response by the sensitivity manipulation in the TS group. Previous research has linked the magnitude of SCR in response to witnessing others in pain with prosocial behavior[2] and implicit race bias[3], where higher SCRs corresponded to increased empathy and prosocial behavior. In conclusion, our findings may suggest a

slight reduction in empathic responses among individuals with TS compared to the control group. However, it is important to acknowledge that the stimuli in our paradigm were presented quite fast, which may have caused overlapping skin conductance responses. In contrast, previous studies examining SCR in empathy for pain paradigms [2,3] used longer inter-trial intervals, and the painful stimuli used were more salient, such as observing hands being pricked by needles or individuals receiving electrical shocks. Overall, it is possible that we missed subtle group differences due to our experimental design. Nonetheless, there was no evidence indicating an increased physiological response to painful situations in the TS group.

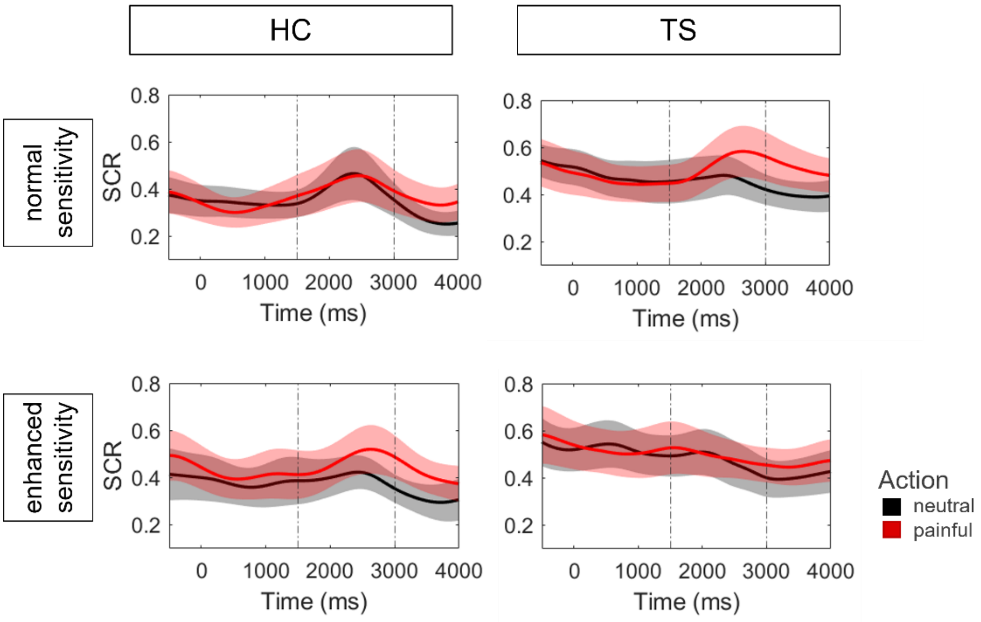

Supplementary figure 1. Skin conductance response in empathy for pain paradigm. Plotted are the time courses of the adjusted phasic driver for the painful (red) and neutral (black) pictures, separately for the controls (HC, left columns) and Tourette patients (TS, right columns) and for normal and enhanced sensitivity. Time-point 0 refers to the picture onset. Statistical analysis is based on our window of interest (1500 - 3000 ms).

References

1. Benedek M, Kaernbach C. A continuous measure of phasic electrodermal activity. *Journal of Neuroscience Methods*. 2010;190(1):80-91.

2. Hein G, Lamm C, Brodbeck C, Singer T. Skin Conductance Response to the Pain of Others Predicts Later Costly Helping. *PLOS ONE*. 2011;6(8):e22759.

3. Forgiarini M, Gallucci M, Maravita A. Racism and the Empathy for Pain on Our Skin. *Frontiers in Psychology*. 2011;2.
